# Patient and dentist perspectives on collecting patient reported outcomes after painful dental procedures in the National Dental PBRN

**DOI:** 10.1186/s12903-024-03931-5

**Published:** 2024-02-07

**Authors:** Elsbeth Kalenderian, Sayali Tungare, Urvi Mehta, Sharmeen Hamid, Rahma Mungia, Alfa-Ibrahim Yansane, David Holmes, Kim Funkhouser, Ana M. Ibarra-Noriega, Janelle Urata, D Brad Rindal, Heiko Spallek, Joel White, Muhammad F Walji

**Affiliations:** 1 Marquette University, School of Dentistry, Milwaukee, WI USA; 2 University of California San Francisco, School of Dentistry, 600 Parnassus Avenue, San Francisco, CA USA; 3 University of Pretoria, School of Dentistry, Pretoria, South Africa; 4 UTHealth School of Dentistry, 7500 Cambridge St. room 4160, Houston, TX TX 77054 USA; 5 UTHealth School of Dentistry, San Antonio, TX USA; 6 FollowApp.Care, London, England; 7 Kaiser Permanente Center for Health Research, 3800 N. Interstate Avenue, Portland, OR 97227-1098 USA; 8 HealthPartners Institute, Bloomington, MN USA; 9 University of Sydney, School of Dentistry, Sydney, Australia

**Keywords:** Patient reported outcomes measures (PROMs), Dental PROMs, Dentistry, Pain, Usability, PROMIS questionnaire, National Dental PBRN

## Abstract

**Background:**

Dental Patient Reported Outcomes (PROs) relate to a dental patient’s subjective experience of their oral health. How practitioners and patients value PROs influences their successful use in practice.

**Methods:**

Semi-structured interviews were conducted with 22 practitioners and 32 patients who provided feedback on using a mobile health (mHealth) platform to collect the pain experience after dental procedures. A themes analysis was conducted to identify implementation barriers and facilitators.

**Results:**

Five themes were uncovered: (1) Sense of Better Care. (2) Tailored Follow-up based on the dental procedure and patient’s pain experience. (3) Effective Messaging and Alerts. (4) Usable Digital Platform. (5) Routine mHealth Integration.

**Conclusion:**

Frequent automated and preferably tailored follow-up messages using an mHealth platform provided a positive care experience for patients, while providers felt it saved them time and effort. Patients thought that the mHealth questionnaires were well-developed and of appropriate length. The mHealth platform itself was perceived as user-friendly by users, and most would like to continue using it.

**Practical implications:**

Patients are prepared to use mobile phones to report their pain experience after dental procedures. Practitioners will be able to close the post-operative communication gap with their patients, with little interruption of their workflow.

**Supplementary Information:**

The online version contains supplementary material available at 10.1186/s12903-024-03931-5.

## Introduction

Currently, over 200,000 active U.S. dental practitioners provide care to more than 156 million patients, expending more than $142 billion annually [1–3]. About 6% of dental outpatient procedures are surgical, [4] and include extractions, implant placements, periodontal treatments, and root canals, with pain the most frequent adverse event (AE) experienced by these patients, accounting for over a quarter (26.2%) of all dental AEs [5]. 

Patient Reported Outcomes (PROs) are reports from patients about their health and are used to better capture the impact of disease interventions. Patient Reported Outcomes Measures (PROMs) are tools or validated questionnaires used to report PROs. PROs measure daily functioning and health outcomes from the patient’s point of view, where condition-specific PROs tend to better record the patient’s state of health. As such, PROs uniquely reflect patient-centered care as they provide specific information on the patient’s response to their treatment, symptoms and in the moment health status [6]. Patient-reported outcomes (PROs) play a crucial role in guiding clinical choices and have become increasingly prevalent in the customization of healthcare and the evaluation of its efficacy [7]. Health information technology (HIT) is frequently employed in the gathering of PROs for these purposes [8–12]. HIT can effectively collect PRO data [13–17] to inform clinical care and promote patient engagement.

Surgical dental procedures are associated with post-operative pain, and poorly managed pain is one of the leading AEs [18–20]. Patient self-report is a critical part of comprehensive pain assessment, [21, 22] given pain’s subjective and multi-dimensional nature [23]. PROs allow clinicians to directly assess patient’s symptoms, symptom burden, functional status, health behaviors, health-related quality of life, and care experiences, [24, 25] and deliver value-based care [26]. Due to the multiple hours-lasting effects of most commonly-used local anesthetic agents, dental patients are unable to predict their pain following dental procedures until many hours later, when they have already returned home, and dental offices are usually closed. This could lead to an over-reliance on pre-emptively prescribed opioids by dental providers because of the difficulty to track their patients’ pain after hours actively. Dentists’ limited ability to collect patients’ pain levels post-operatively may lead to early opioid prescriptions despite addiction risk and inferior post-op pain relief compared to non-opioids, [27–36] to safeguard against worst case scenarios and/or patient dissatisfaction from misconceptions about opioids [32, 37, 38]. 

Here we report on the patient and dentist perspective as part of a pilot study that implemented an mHealth platform for conducting follow-ups after dental procedures to assess patients’ post-operative dental pain experience [39]. Using the mHealth platform, we collected pain-related data from patients who had undergone at least one surgical dental procedure associated with post-operative dental pain specified in the afore mentioned published study protocol, over twenty one days after the procedure and fed this information back to the dentist. Patients received automated text messages on days 1, 3, 5, 7, 14, and 21 after their dental procedure with an electronic link, which they used to report their PROs. Information on patients’ dental pain experience in terms of the severity of pain experienced on a scale of 0–10; the extent to which pain interfered with their daily activities such as speaking, eating, walking, and sleeping; the medications that they were consuming to manage the pain; whether they had any complications such as post-operative bleeding and swelling were among the PROs that were collected and shared with the dentists. As a result, dentists were alerted when patients experienced severe pain or had any post-operative complications or had questions and the platform was thus used to communicate with the patient rapidly. This pilot study was developed, as part of a larger study, to explore how dental practitioners and patients would perceive dental PROs captured using mobile phones to improve post-operative dental communication after a painful dental procedure. Since mobile phones are readily available to most patients, it provides a convenient medium to bridge the communication gap and increase patient access to their practitioners while simultaneously creating opportunities for practitioners to improve the quality of patient care by alleviating patient suffering, preventing or minimizing severe post-operative complications, and reducing the use of opioids as the first line of treatment. It also has the potential benefit of reducing or eliminating unnecessary and unscheduled office visits or disruptive phone calls from patients after hours. Here we report on an in-depth thematic content analysis of the PRO data to better understand the barriers and facilitators as perceived by patients and dentists [40]. 

## Methods

The pilot study was conducted as preparatory work for a larger observational study [39] seeking to characterize post-operative dental pain by recruiting dentists and patients in the National Dental Practice-Based Research Network (National Dental PBRN) setting [41–43]. The National Dental PBRN is a collective network of dental practices that includes private and group practices, public health clinics, community health centers and Federal Qualified Health Centers (FQHCs), academic institutional settings, and special patient populations [44–46]. The pilot included (1) a usability session in which patients and dentists viewed a simulated scenario of the platform, (2) feedback after using the platform in real clinical practice. Informed consent was obtained from all subjects. Criteria from the COREQ checklist have been used to guide the reporting of this study.

### Data collection

The study’s Principal Investigator (PI), author MW was the interviewer for all the practitioner interviews and patient usability sessions. One of the research coordinators, authors AIN, SH and JU was the facilitator. The patient interviews after using the platform were conducted by either MW, AIN, SH or JU. The PI has more than 15 years of experience in conducting mixed-method implementation research that involves interviewing participants for usability testing and acceptance of technological interventions. The research coordinators were trained by the PI in conducting these interviews by initially facilitating and then conducting mock interviews.

Practitioners and their patients who had participated in the observational study were purposefully sampled to participate in the interviews based on several criteria including their use of the mHealth platform, any functionality-related issues faced while using the platform, ability to participate in an online interview, gender, age and geographic location to ensure inclusion and representation. All participants were recruited through the National Dental PBRN. Coordinators identified eligible practitioners for the interviews, directing them to the Principal Investigator (PI) for details and consent procedures. Using the snowball sampling technique, the practitioners identified potential patient participants, who were also referred to the PI for the interview. All interviewees were recruited through either email or text messaging, reminders were sent in case of no response. Of the 26 dentists and 50 patients contacted, 4 dentists and 19 patients did not respond to the interview invitation after 3 attempts of contacting them. 1 patient declined to participate without providing a reason, and 2 patients did not show up for the scheduled interview. After informed consent was obtained, the research coordinators scheduled the interview. The interviewees had no prior interaction with their interviewer. On the day of the interview, the interviewer and facilitator (if present) introduced themselves and provided a brief summary of the research project to the interviewees prior to commencing the interview. All participants were remunerated for their participation after completing the interview.

The data for this research was collected through semi-structured interviews, which were guided by questions about current follow-up practices after dental visits; assessing the usability of the mHealth platform; identifying the preferred features of such an mHealth platform; assessing the structure of the follow-up mHealth questionnaires; gauging the acceptance and implementation of the mHealth platform for follow-up into routine dental practice. The interview guides were developed collectively by the research team. Recognizing the distinct perspectives and experiences of dentists and patients, we crafted separate but complementary sets of questions. For patients, the guide focused on the user interface, accessibility, and personal relevance of the platform, probing their comfort with technology and how the platform met, or failed to meet, their healthcare communication needs. Conversely, for dentists, the guide delved into the practicality of the platform in clinical settings, its efficacy in enhancing patient care, and its integration with existing dental practice workflows. The guides were iteratively refined, ensuring they were comprehensive yet concise enough to encourage candid feedback.Virtual semi-structured interviews with 22 dentists and 32 dental patients were conducted by one of 4 study team members during 2021 and 2022. Each interview duration ranged between 25 min and 1 h. In addition, audio data were recorded and transcribed. See Appendix 1 for the semi-structured interview questions.

### Data analysis

Independent free-coding of the transcripts was performed by 3 study team members for 4 interviews (2 patient interviews and 2 dental provider interviews) by twice reading the transcripts and listening to the audio recordings and develop a primary coding framework. Variations in coding were discussed for consensus validation and code definitions were assigned before coding the other interview transcripts. Each of the remaining 50 interviews was coded by 2 of the 3 study members independently to ensure methodological rigor. New codes were identified, or primary codes were modified as necessary throughout this process to create an inclusive and comprehensive set of codes. After all interviews were coded, any variations in coding were discussed between the 3 coders before analysis. Codes were assessed for usage and co-occurrence. Inductive strategies were used for the thematic analysis of the qualitative data [47, 48]. Codes and themes were discussed with the research team, alternate interpretations were explored, and themes were revised if necessary. Google Docs was used to code the transcripts and Microsoft Excel was used to create and maintain the coding framework used to organize the quotes, assign respective codes and conduct qualitative analysis.

## Results

### Patient characteristics

The mean age of the 22 dentists was 48.73 (SD: +/- 9.39) years and the 32 patients was 41.16 (SD: +/- 14.69) years. Adult participants older than 18 years old were included in this study. Both dentists and patients were recruited from the six different geographic regions of the United States – Northeast, South Atlantic, Midwest, Southcentral, Southwest, and Western regions. Table 1 below shows the distribution of the participants who were interviewed.

**Table 1:** Summary of Participants characteristics

	Dentist participants *N* = 22	Patient participants *N* = 32
Age range, years		
18–35	1	14
36–50	12	10
51–65	8	6
> 66	1	2
Sex		
Male	9	14
Female	13	18
Race		
Asian	8	12
Black or African American	1	3
Middle Eastern	1	0
White or Caucasian	10	14
More than one race	1	2
Other/Prefer not to answer	1	1
Ethnicity		
Hispanic or Latino	1	2
Not Hispanic or Latino	21	30
United States Region		
Northeast	2	3
South Atlantic	2	2
Midwest	3	3
Southcentral	2	1
Southwest	12	21
Western	1	2

### Theme analysis

The mHealth platform was deemed a useful tool for dental post-procedural follow-up and patients and dentists perceived multiple features as desirable. Table 2 shows the major themes identified.

**Table 2:** Themes of likes and dislikes of using the mHealth platform

Themes
1. **Sense of Better Care**: Frequent automated follow-ups provided a sense of better care and positive patient experience while being time and effort saving for the dentists
2. **Tailored Follow-up**: Preference for tailored follow-up duration based on dental procedure and patient’s pain experience
3. **Effective Messaging and Alerts**: ‘Messaging’ and ‘Alerts’ features perceived as most helpful
4. **Usable Digital Platform**: Concise and comprehensive questions on a user-friendly platform which was perceived as acceptable in digitally-challenged groups as well
5. **Routine mHealth Integration**: Preference to routinely use mHealth platform for post-operative follow-up with suggested additional features

#### Sense of better care

Patients felt a better sense of care and expressed feeling cared for by their dentist because of the regular multiple automated follow-up messages sent during the follow-up period. Dentists also thought that the continuous follow-up communication provided a positive patient experience and aided in being more connected to their patients.

*“I think it’s like relationship building, practice building because they realize we care. We want to make sure that they’re healing well and that they’re comfortable. So, I think it was a very positive thing”* – Dentist 22, Female, 41 years old.

*“I think that I like that I’m being cared for, like beyond the office, beyond the premise of the office. I think that it (constant follow-up) kind of shows that my provider really does care for the way that I’m feeling.”* – Patient 11, Female, 20 years old.

*“I think it it’s a nice way to show that although automated, it shows that the clinic or the provider is concerned about the overall well-being of the patient after the procedure.”* – Patient 3, Male, 40 years old.

Dentists acknowledged that electronic follow-up through automated questionnaires might not be equivalent to personal interaction with the patient. Still, it helps the dentist overcome the challenge of lack of time they have for making follow-up calls and ensures timely follow-up.

*“First and foremost, I liked how it’s automated, so it’s not something where I have to do it myself on the other hand, it certainly doesn’t replace that personal interaction that one would get if I called them by phone. But oftentimes you just don’t have that kind of time to devote for those follow up calls.”*– Dentist 3, Male, 44 years old.

*“I think at first, it felt, I don’t know if nurturing is the word, but that you were being attended to after a procedure. And I think everybody wants to feel that way. But that’s a perfect world, right? Everyone’s doctor can’t check up on you after the procedure. But that’s what it felt like, kind of like an extension of the doctor’s care in a way…it is nice to know that they’re still showing concern…”*– Patient 21, Female, 47 years old.

Patients mentioned that through the platform, their dentist communicated promptly and provided the dentists an option to communicate at their convenience. Patients were cognizant of the time and effort it takes in follow-up and mentioned that they didn’t mind the supposed ‘lack of personalness’ of follow-up through the platform.

*“It’s a good way of communicating without having to take a lot of time because I could fill the form out. If he needed to get back with me pretty quickly, he could do that. So and that’s the way the world is going now. And so, I didn’t mind the impersonalness of it. I thought, wow, what a time saving way to communicate with your physician.”*– Patient 28, Male, 67 years old.

We conclude that frequent automated follow-ups provided a sense of better care and positive care experience for the patient participants while at the same time being perceived by the providers as saving time and effort.

#### Tailored follow-up

In this pilot study, follow-up messaging was done on days 1,3,5,7,14 and 21. The views on frequency and duration of follow-up were divided; some participants found it adequate, while others mentioned that it should be tailored to each patient.

*“In real clinical situation, I don’t think we’re going to do that many post-ops (7 check-ins across 21 days), you know, really, unless they have continual issue, then we tell them to, you know, to contact us, instead of keep reaching out, because it gets kind of overwhelming to patients when you start to over communicate very much.”* – Dentist 9, Female, 56 years old.

*“I would assume that starting at day seven, I think it’s very likely that somebody would respond to the seven-day response. And I think that the 14th day will be less likely and the twenty first day will be even less likely than that. I think that you’ll see a drop off because at that time the person feels like their needs have been met and then they might not be so inclined to give information because their needs are met.”* – Patient 14, Male, 28 years old.

Several provider participants suggested that a week of post-operative follow-up would be ideal. However, many participants mentioned that patients may not respond or may opt out after 7 days once their needs have been met.

*“I think the number of messages, or the frequency of the messages should be related to the procedure that is being done and the expected recovery time. I’ve had wisdom teeth pulled out, so I’d probably expect something to be, you know, five days or five to seven days or something like that. Even a crown that I’ve had performed on me probably five to seven days. I think going beyond 21 days, I think would just I would just opt out at that point and probably opt out after day seven.”* – Patient 3, Male, 40 years old.

*“Obviously (procedure-specific follow-up duration is needed) for extractions, deeper fillings, and sometimes deeper crowns. But implants, definitely that would be nice to track because everyone responds differently, especially later on, we will have that inflammatory response in a couple of weeks.”* – Dentist 11, Female, 47 years-old.

We conclude that there is a preference for temporal and tailored follow-up based on the specific dental procedure and the patient’s pain experience in real time. Most dental procedures would likely require follow-up for up to 7 days.

#### Effective messaging and alerts

The platform had a text entry feature at the end of the questionnaire where a patient could ask their dentist any questions or express concerns, which were sent as an alert to the dentist via text message on their cellphone. Both dentists and patients perceived the ‘messaging’ feature of the mHealth platform as useful and helpful because it facilitates quick communication, provides an opportunity to interact directly with each other, and enables prompt delivery of care.

*“I think that the concept of how patients respond and the feedback going back and forth is fantastic, and you really hit the nail on the head with that one.”* – Dentist 17, Male, 67 years old.

*“I think that it’s pretty helpful just to be able to communicate with my provider without having to, like, call and, you know, talk to the front desk and then get to my provider and then their email and then everything that goes with all those steps. And this is just like a good link, a good bridge between me and my provider. And then they just get back to me whenever they can.”* – Patient 11, Female, 20 years old.

*“I had numerous patients that probably would have called the office numerous times, and instead they could just send me a little text message. And it was very convenient because it just pops right up and I could get back to them, you know, almost in real time or at my convenience if I was extraordinarily busy. And then. You know, avoid needing to wait for the receptionist to send a message to me or to have me have a free time to actually call the patient, or it was just it was a lot easier.”* – Dentist 13, Female, 41 years old.

*“I felt comfortable with it (follow-up through chat feature/text communication), because a lot of times they’re busy, and they don’t really call back very fast. So, the speed of the answer, I guess, through text, made it great, because I could assume it’s probably helpful for them in between patients to maybe just shoot you back a simple answer”* – Patient 21, Female, 47 years old.

We conclude that the “Messaging” and “Alerts” features of the platform were perceived as most helpful by providers and patients alike. Rather than having the providers receive all patient responses, triggered messages based on a certain threshold for increased pain, swelling, or bleeding were perceived as optimal.

#### Usable digital platform

Patients perceived the mHealth questionnaire as intuitive, straightforward, with clear questions, and not too long.

*“I thought it (the follow-up questionnaire) was spot on because it stayed focused on the pain you were experiencing and whether that pain is growing or diminishing or staying the same and what medications you’re on. So, I thought it was simple, short, and to the point.”* – Patient 28, Male, 67 years old.

Both patients and dentists found the current mHealth platform was user-friendly, easy to navigate, and one that could be accepted by technologically digitally challenged groups. In addition, dentists liked the layout of the system, where they could view all patient responses, compare the patient’s pain experience across the follow-up duration, and communicate through the system with their patients at the same time.

*“It’s user-friendly, it’s not it’s not too clumped together. I can just go into anyone, click it and able to get the relevant information I need, it’s very clearly stated the days and on the other side, the conversations, what was done, the mobile communication. And all the statistics, whether, you know, the patient responds when they respond, and I especially like the numbering system, the pain and also swelling. Yeah, it’s very elaborate, but at the same time very navigable.”* – Dentist 4, Male, 48 years old.

While patients also thought the system was easy to navigate, they specifically appreciated that there was no need to download any phone application or sign in to the system. Patients perceived that the questionnaires in the platform were easy to access once they clicked the link received through the text message and liked that the questions opened in a web browser which enabled quick and direct access and answering of the questions, making it time-efficient and user-friendly.

*“This (overall experience using electronic follow-up system) was really easy. You’re getting the text message instead of having to look it up on an email or download an app or something. It was so much easier to do. So, it was great not having to do a lot, just clicking on that and then just answering the questions and sending. It was awesome.”* – Patient 22, Female, 42 years-old.

*“So, I’m 58 and I am not… I share that only because it’s no secret that older people struggle with technology, right? So, it’s usually not as intuitive for me as it would be a 20-year-old for example, but I thought this app was very user friendly and very easy to use, and no question, all of it worked beautifully, there were no glitches, there were no delays. It was very easy, quick, it was very nice.”* – Patient 30, Female, 58 years-old.

*“We actually did not have super young patients. I think the youngest one of those five, were maybe in their forties, fifties. So, so in terms of the digital literacy, I think they were able to manipulate the system pretty well. One patient, he actually was 80 years old so it’s not a young person medically acknowledged so well, and for him, he did not have any other issues. So, he was able to fill in (the PRO forms/surveys). I looked at his pain score he put in, so it looks like it worked well for him.”* – Dentist 15, Female, 42 years old.

We conclude that the mHealth questionnaire was developed with concise and comprehensive questions on a user-friendly platform which was perceived as acceptable by all participants, including those who were deemed potentially digitally-challenged.

#### Routine mHealth integration

Both dentists and patients preferred using a mHealth platform for the follow-up process. Multiple reasons for preference in using the platform were mentioned - some liked the ability to send messages the most, dentists liked it because of the notifications that were generated and because of the ability to monitor and compare patient’s symptoms and pain experience, while other participants liked it because it kept post-operative instructions documented.

*“I personally would prefer the mHealth platform because I would much rather text and get stuff done that way than picking up the phone, looking for the phone number and calling and writing notes down because that’s just how I work. But I would much rather text and have it in my phone and be able to know, ‘Oh, okay she replied back. This is what she said. And then she said, I could come in on April 15th.’ So, I would prefer that way moving forward.”* – Patient 25, Female, 56 years old.

We conclude that providers and patients alike were enthusiastic about incorporating mHealth for routine follow-up of patients, especially after extensive or potentially painful dental procedures.

#### Additional mHealth platform suggestions

Participants offered many ideas to further advance the mHealth platform to improve the user experience. The incorporation of an AI system to answer common patient questions or in-text responses while typing a message, as well as the ability to schedule a phone/video call with their provider on the platform were suggested to further improve the patient care experience.

*“But something else I’m doing a lot with my family, especially with COVID, is video chat. So even if the doctor or their assistant had time to video chat, that could be pretty cool. So, I mean, you can add it in the app and maybe… I’m thinking like Teladoc visits because not only asking about the pain, but they can also look at it, I guess kind of hold your phone up and see how they’re doing. I don’t know.”* – Patient 26, Female, 34 years old.

*“I don’t know if in the future you’re probably going to implement a system where this app will be able to also communicate with that office’s software… and the patients will be able to see like when there are dates (for scheduling follow-up) and stuff like that.”* – Patient 6, Male, 31 years old.

Patients suggested incorporating resources within the platform that provide post-operative instructions and pain management guidance.

*“We might get on Google and try to find out what pain my level should be. We might forget what the doctor told us. Maybe having some information about the procedure that you had and something that you can read and say, this is what’s expected, this is normal. And if you’re experiencing something that’s not normal, this is the warning signs because I have no idea if what I went through was normal or not or if I should alert someone. So that would be helpful.”* – Patient 26, Female, 34 years old.

Integration with the existing provider’s EHR was also mentioned as an important feature.

*“If this could be integrated into the dental record system, so this way everything would be on the same platform. So, that would be probably the best.”*– Dentist 15, Female, 42 years old.

We conclude that the patient and provider participants had many exciting suggestions for further expanding the mHealth platform’s functionality to further improve its users’ experience.

## Discussion

Both patients and dentists reported positive experiences using “frequent automated follow-ups” in managing the patient’s post-operative journey [49]. Patients felt cared for and connected to their dentist, and dentists were able to provide timely follow-up without having to make time-consuming phone calls. Although the follow-up messages were not as personal as direct communication, patients appreciated the convenience and time-saving aspect of the automated system. The point of view of our study provider participants is shared with other practitioners, who acknowledge the benefits but also recognize the training and information barriers we managed to overcome [50]. In fact, many barriers persist to the implementation of PROMs in routine care, including the use of technology, competing demands, workflow issues, unclear rationale, and unclear execution [51]. 

We also found mixed views on the frequency and duration of follow-up, suggesting the need for “tailored follow-ups”. Some participants found the provided schedule adequate, while others suggested tailoring follow-up to each patient’s needs. Providers suggested a week of post-operative follow-up would be ideal, while patients may opt-out after seven days once their needs are met. Procedure-specific follow-up duration was also suggested, with preferences varying based on the type of procedure and the patient’s pain experience.

The mHealth platform had a messaging feature where patients could communicate with their dentist via text message, and both dentists and patients found this feature useful for quick communication and prompt delivery of care. Dentists appreciated the convenience of responding to patients’ concerns almost in real time. In contrast, patients appreciated the speed of the response and the ability to communicate with their provider without having to go through several steps. Additionally, the platform had a trigger for swelling and pain that dentists found helpful in contacting the patient. The theme of concise and comprehensive questionnaires reinforces the notion that routine use of well-developed, validated PROMs enhances communication between provider and patient, improves the patient’s care experience and outcome, and may provide information for value-based system improvements [52]. A recent Cochrane review summarized PROMs to produce a moderate improvement in diagnosis, record keeping, and disease control and a small improvement in quality of life [53]. 

Our results demonstrated that patients and dentists found the mHealth platform to be user-friendly and easy to navigate, with clear and concise questions. Patients appreciated the convenience of not having to download an app, and found it time-efficient. Even older patients were able to use the system effectively, indicating that it was acceptable for individuals with limited technology experience. Overall, the mHealth questionnaire was considered well-designed and perceived positively by participants. mHealth applications to measure patient reported outcomes (PROs) and ePROMs remain a fairly novel application in medicine [54, 55]. Importantly, the way providers will use PROMs is, at least in part, shaped by their relationship with their patients, [56] which will influence the development of ePROMs using an mHealth platform. Implementation of mHealth and PROMs in practice can be facilitated with good leadership support and a culture of learning [51]. The use of implementation science frameworks may be useful in addressing major barriers, although lack of time appears to be a difficult barrier to overcome [51, 57]. 

In order to make PROs useful, PROs should be incorporated into the daily clinic workflow. E.g., by incorporating PROs into the standard care visit, PROs and PROMs facilitate the dialogue between the practitioner and patient leading to better shared decision making and patient-centered care. Providers agree that PROs enhance patient engagement and shared decision-making when integrated into clinical care, as PROMs allow for an assessment of the patient’s own experience of their health condition, ideals, preferences, and health care goals [58]. However, putting them into practice proves difficult [59, 60]. Work flow issues, data overload, staff burden, and potential increased clinic visit times are all logistical issues that stand in the way [58]. Importantly, integrating the PRO data into the EHR has to be one of the top issues for advancing PROs into clinical care. Most EHR are not yet well enough developed to accept PROM data and conversely PROM platforms do not easily integrate with EHRs. This is likely at the root of other technical issues, specifically how to make PROMs actionable for patient care [61]. Better dashboards, reminders, and PRO-triggered automated scheduling are technical issues that would turn PROM data into patient-centered action items [58, 62]. Clearly, as done in this pilot study, a user-centered design is key in developing ePROMs that are highly functional for both practitioner and patients [63]. 

The findings of the study will be used directly to further refine the mHealth platform and relevant patient reported outcome measures (PROMs) before the implementation of the system in a large national observational study of 150 dental providers and up to 3147 patients who will receive push notifications through text messages on their mobile phones at designated time intervals following dental procedures [39]. Through patients’ use of their mobile phones, we expect to identify specific pain levels and other issues after surgical dental procedures. The study’s primary outcome will be the patients’ reported pain experiences. Secondary outcomes include pain management strategies and medications implemented by the patient and provider and perceptions of usefulness and ease of use by patients and providers.

Our study results were limited by the characteristics of the sample who participated. The dentists who volunteered to participate may have been more interested in technology approaches. In this study, participants either were exposed to the platform in a simulated usability setting or used for a short time in practice as part of the pilot. It is possible that the perspectives of dentists and patients may change after using the mHealth platform for an extensive period in dental practice, or we may have discovered different perspectives from a larger and more representative sample.

While our study was focused on receiving feedback about the mHealth platform for postoperative dental pain, it is likely that the themes we identified are also relevant to other healthcare settings. A systematic review on adoption of m-Health by healthcare professionals found similar characteristics such as perceived ease of use and interaction/communication between patient and provider through the mHealth platform as facilitators for mHealth adoption [13]. Multiple studies advocate interoperability and integration of mHealth platforms with electronic health records as a future direction that would increase the adoption of mHealth in healthcare settings [13, 64, 65]. 

## Conclusion

We engaged end-users (practitioners and patients) in the assessment of the design features of a mobile phone platform to be used for the implementation of PROMs. We conclude that frequent automated, and preferably tailored follow-up messages using an mHealth platform, provided a positive care experience for the patients participants, while the provider participants felt it saved them time and effort. Patients thought that the mHealth questionnaires were well-developed and of appropriate length. The mHealth platform itself was perceived as user-friendly by all types of users, and most would like to continue using it.

### Electronic supplementary material

Below is the link to the electronic supplementary material.

**Supplementary Material 1:** Provider and patient semi-structured interview questions

## Data Availability

The datasets generated and/or analyzed during the current study are not publicly available as they consist of transcripts that convey the thoughts and opinions of the providers and patients that were interviewed. Informed consent was obtained for using these data as part of the specific study only and not for wider sharing or distribution. Fully deidentified data are however available from the corresponding author upon reasonable request.
