# Supplementary material for: Patient and dentist perspectives on collecting patient reported outcomes after painful dental procedures in the National Dental PBRN
Source: BMC Oral Health. 2024 Feb 7;24:201. doi: 10.1186/s12903-024-03931-5 (PMC10848340; doi:10.1186/s12903-024-03931-5)
Supplement: Supplementary file 1 — Supplementary Material 1: Provider and patient semi-structured interview questions [file 12903_2024_3931_MOESM1_ESM.docx]

APPENDIX 1

PROVIDER SEMI -STRUCTURED QUESTIONS

1. What did you like about the platform?
2. What did you not like?
3. What can we improve/What would you like to add?
4. How do you see the tool working with your regular daily schedule
5. How do you usually contact your patients? Cellphone? Home phone? Text? Email? Who usually contacts them?
6. What are the current procedures followed at your clinic when a patient has pain after a dental procedure?
7. If the patient calls/text/emails you/your staff with a complaint of post-op pain, what do you do? Do you ask for their pain level, diagnose them, and prescribe medication?
8. How do you document this post-op pain interaction in the EHR? Do you document it at all? Who documents it?
9. Did you receive any alerts once patients completed the surveys?
   1. If yes, how did that go?
10. What do you think about the system in general as a communication tool with your patients?
11. Would you want to continue with the platform after the study?
12. Do you have any other questions or comments?

PATIENT SEMI -STRUCTURED QUESTIONS

1. What did you like about the platform?
2. What did you not like?
3. What can we improve?
4. Do you see this working as part of your dental care?
5. What do you think about how often surveys were sent to you?
6. What about the length of the survey?
7. How easy was it to answer the question about pain?
8. What are your expectations of how your dentists may communicate back to you?
9. Let's say your dentist didn't come back to you after 4-6 hours. What would you do then?
10. Would you like to continue to use the platform when seeing the dentist?
11. Do you have any suggestions or comments?
